# In this month’s *Bulletin*

**DOI:** 10.2471/BLT.16.000616

**Published:** 2016-06-01

In the editorial section, Anthony Costello et al. (406) announce WHO’s collaborative effort to define the syndrome associated with congenital Zika virus infection. Islene Araujo de Carvalho et al. (407) call for papers for a special issue on innovation for healthy ageing. Michel Sidibé (408) points to an opportunity to secure funding for the next stage of the response to the human immunodeficiency virus epidemic.

Menelaos Tzafalias (411–412) reports from Greece on the fake lifejackets sold to refugees for their sea crossings to Europe. Richard Peto tells Andréia Azevedo Soares (413–414) why WHO should focus on the big causes of death from noncommunicable diseases.

## Botswana, South Africa & Zambia

## Are infection control measures working?

Andre R Verani et al. (415–423) review legislation designed to reduce tuberculosis transmission.

## Gambia

## A picture of the whole population

Ifedayo MO Adetifa et al. (433–441) report tuberculosis prevalence from a nationwide survey.

## Ghana

## Why are babies missing out?

Maureen O’Leary et al. (442–451) investigate delays in vaccination of low-birth-weight infants. 

## Malawi

## Preventing malaria

Emmanuel Chanda et al. (475–480) describe efforts to scale up vector control programmes.

## Mexico 

## Care in the childbearing year

Ileana Heredia-Pi et al. (452–461) measure the adequacy of antenatal care.

## Global

## Mopping up after Ebola

Kelly L Edmunds et al. (424–432) present ways to deal with contaminated waste.

## Working towards universal health coverage

Kalipso Chalkidou et al. (462–467) argue that explicit ways of setting priorities are needed**.**

## Making a convincing case for investment 

Ian Anderson et al. (468–474) list the persuasive skills needed by health ministries.

